# Comparable frequency of *BRCA1, BRCA2 *and *TP53 *germline mutations in a multi-ethnic Asian cohort suggests *TP53 *screening should be offered together with BRCA1/2 screening to early-onset breast cancer patients

**DOI:** 10.1186/bcr3172

**Published:** 2012-04-16

**Authors:** Daphne SC Lee, Sook-Yee Yoon, Lai Meng Looi, Peter Kang, In Nee Kang, Kavitta Sivanandan, Hany Ariffin, Meow Keong Thong, Kin Fah Chin, Nur Aishah Mohd Taib, Cheng-Har Yip, Soo-Hwang Teo

**Affiliations:** 1 Cancer Research Initiatives Foundation, Sime Darby Medical Centre, 1 Jalan SS12/1A, Subang Jaya, 47500 Selangor, Malaysia; 2 Department of Pathology, University Malaya Medical Centre, University Malaya, Kuala Lumpur, Malaysia; 3 University Malaya Cancer Research Institute, University Malaya Medical Centre, University Malaya, Kuala Lumpur, Malaysia; 4 Department of Paediatrics, University Malaya Medical Centre, University Malaya, Kuala Lumpur, Malaysia; 5 Department of Surgery, University Malaya Medical Centre, University Malaya, Kuala Lumpur, Malaysia

## Abstract

**Introduction:**

Germline *TP53 *mutations cause an increased risk to early-onset breast cancer in Li-Fraumeni syndrome (LFS) families and the majority of carriers identified through breast cancer cohorts have LFS or Li-Fraumeni-like (LFL) features. However, in Asia and in many low resource settings, it is challenging to obtain accurate family history and we, therefore, sought to determine whether the presence of early-onset breast cancer is an appropriate selection criteria for germline *TP53 *testing.

**Methods:**

A total of 100 patients with early-onset breast cancer (≤ 35 years) treated at University Malaya Medical Centre between 2003 and 2009, were analyzed for germline mutations in *BRCA1, BRCA2 *and *TP53 *by full DNA sequencing. Of the mutations identified, we examined their likely pathogenicity on the basis of prevalence in a case-control cohort, co-segregation analyses and loss of heterozygosity (LOH) in tumor tissues.

**Results:**

We identified 11 *BRCA1 *(11%) and 6 *BRCA2 *(6%) germline carriers among early-onset breast cancer patients. Of the 83 BRCA-negative patients, we identified four exonic variants and three intronic variants in *TP53*. Of these, two exonic variants are clinically relevant (E346X and p. G334_R335dup6) and two novel missense mutations (A138V and E285K) are likely to be clinically relevant, on the basis of co-segregation and loss of heterozygosity (LOH). Notably, E285K was found in two unrelated individuals and haplotype analyses suggest a founder effect. Two of the three intronic variants are likely benign based on their prevalence in a control population. Clinically relevant *TP53 *germline mutations were identified in three of the four patients (75%) with a family history of at least two LFS-linked cancers (breast, bone or soft tissue sarcoma, brain tumors or adrenocortical cancer); 1 of the 17 patients (6%) with a family history of breast cancer only, and 1 of the 62 patients (< 2%) with no family history of breast or LFS-linked cancers.

**Conclusions:**

Our study reports germline *BRCA1, BRCA2 *and *TP53 *mutations are found in early-onset breast cancer patients at 11%, 6% and 5% respectively, suggesting that *TP53 *mutation screening should be considered for these patients. However, we find that even in low resource Asian settings where family history is poorly reported, germline *TP53 *mutations are found predominantly among breast cancer patients with a family history of LFS-linked cancers.

## Introduction

To date, germline mutations in at least 10 genes linked to DNA repair have been shown to be associated with an inherited risk for breast cancer [1]. This includes *TP53*, a tumor suppressor gene that plays a pivotal role in the cell's response to DNA damage by inducing pathways involved in apoptosis, cell cycle arrest and the DNA repair mechanism in order to maintain the cell's genomic integrity [2].

Although rare, germline mutations in *TP53 *are associated with a significantly increased risk of female breast cancer, bone or soft tissue sarcomas, brain tumors and adrenocortical carcinomas (ACC) [3-7]. To date, the majority of studies have examined the prevalence of *TP53 *germline mutations among families identified through the genetics clinics and these studies have shown that the majority of families with germline *TP53 *mutations fulfill either the original Li-Fraumeni syndrome (LFS) criteria, Li-Fraumeni-like (LFL) criteria or the Chompret criteria and are present with a family history of at least one of the four LFS-linked cancers (breast, bone or soft tissue sarcomas, brain tumors and ACC) [1,5,8,9]. More recently, population-based studies have found *TP53 *germline mutations among women with early-onset breast cancer aged 40 years and below [10,11].

However, the reliance on family history and recruitment through the genetics clinics poses significant challenges in many Asian countries, particularly low and middle income countries such as Malaysia. Clinical genetics services are relatively new and underdeveloped and, moreover, our data and data from Asian Americans have shown that risk assessment models for *BRCA1 *and *BRCA2*, which were largely built on families with multiple cancers, underestimate the number of *BRCA *carriers in Asians compared to Caucasians [12,13]. We have previously found that family history is poorly reported in Asian families in Malaysia, in part due to the stigma associated with cancer in Asian families, significant geographical dispersal of families and the lack of robust cancer registries [12,14]. There is currently no data in any Asian cohort to determine whether this underestimation of number of carrier families also applies to other genes.

In order to determine the significance of *TP53 *germline mutations to breast cancer risk in our multi-ethnic cohort, we sought to determine the prevalence of *TP53 *germline mutations in a hospital-based cohort of Asian breast cancer patients diagnosed before age 35 years. Of the mutations identified, we examined their likely pathogenicity on the basis of prevalence in a case-control cohort, co-segregation analyses and loss of heterozygosity (LOH) studies in tumor tissues.

## Materials and methods

### Cohort selection

A total of 1,060 breast cancer patients, who were treated at University Malaya Medical Centre, Kuala Lumpur between January 2003 and December 2009, were recruited into the Malaysian Breast Cancer Genetics Study (MyBrCa). All patients gave informed consent and the study was approved by the University Malaya Medical Centre ethics committee.

*BRCA1 *and *BRCA2 *sequencing and large chromosomal deletion analysis was conducted in all patients diagnosed with invasive breast cancer aged below 35 years and those with a family history of breast and/or ovarian cancer [12,15]. Of the BRCA-negative patients, we screened for germline *TP53 *mutations in all 83 women who developed invasive breast cancer before 35 years of age, regardless of family history.

### Mutation detection

Genomic DNA isolation and DNA sequencing was conducted as previously described [15]. Full DNA sequencing was carried out in both directions on the entire *TP53 *coding region and intron-exon boundaries, and annotated using HGVS nomenclature with the GenBank reference sequence NC_000017.9.

### Case-control studies

Genotyping analyses of the seven mutations identified in this study were conducted using the Sequenom MassARRAY platform (San Diego, CA, USA) on 880 women (181 Malays, 530 Chinese, 169 Indians) with invasive breast cancer and on 270 female controls (90 Malays, 90 Chinese, 90 Indians). All mutations identified were confirmed by DNA sequencing.

### Haplotyping analyses

Four *TP53 *microsatellite markers were used as previously described: p53CA, an intragenic dinucleotide marker; VNTR, a marker with a pentanucleotide repeat in intron 1 [16] and two markers flanking the *TP53 *gene, D17S786 and D17S796 [17].

### Loss of heterozygosity (LOH) analyses

Where available, 15 to 20 μM of formalin fixed paraffin-embedded (FFPE) specimens were retrieved for DNA analyses. Each sample had approximately 40 to 80% tumor cells and genomic DNA was isolated using the QIAamp DNA Mini Kit (Qiagen, Hilden, Germany) according to the manufacturer's protocol. LOH analyses were conducted by DNA sequencing and genotyped using the Sequenom MassARRAY platform (San Diego, CA, USA).

## Results

In our ongoing study of breast cancer predisposition genes in a hospital-based cohort of breast cancer patients, we have analyzed *BRCA1 *and *BRCA2 *in all patients younger than 35 years diagnosed with invasive breast cancer. Between January 2003 and December 2009, 281 patients were diagnosed with invasive breast cancer before age 35 years at the University Malaya Medical Centre, Kuala Lumpur and 100 patients were recruited into the MyBrCa study. Of these, 11 (11%) and 6 (6%) were found to have deleterious mutations in *BRCA1 *and *BRCA2 *respectively.

We have analyzed germline *TP53 *mutations in the remaining 83 individuals who tested negative for both *BRCA1 *and *BRCA2 *mutations (Table 1). Of these patients, 4 (4.8%) had a family history of at least one case of the other LFS-linked cancers (bone or soft tissue sarcoma, brain tumors or ACC), 17 (20.5%) had a family history of breast cancer only, and the remaining 62 had no family history of any of the LFS-linked cancers. Mutation analyses have revealed four unique exonic germline mutations; of which one (E285K) was found in two unrelated individuals, and three unique intronic germline mutations; of which one (c.74 + 14 T > C) was found in two unrelated individuals (Table 2).

**Table 1:** Characteristics of families recruited into the *TP53 *study (*n *= 83)

Characteristics	No	(%)
**Ethnicity**		
Malay	29	35
Chinese	44	53
Indian	10	12
**Family history (1° and 2° relatives)**		
1 case of breast cancer	13	16
2 cases of breast cancer	3	4
≥ 3 cases of breast cancer	1	1
No family history of breast and/or ovarian cancer	62	75
≥ 1 case of LFS-linked cancers	4	5

Table 1 shows the characteristics of 83 BRCA-negative early-onset breast cancer patients analyzed for germline ***TP53 ***mutations according to their ethnicity, personal and/or family history of breast cancer in first- and second-degree relatives and the presence of LFS-linked cancers (breast cancer, bone and soft tissue sarcoma, brain tumors, adrenocortical carcinoma (ACC)) in the families.

**Table 2:** Germline *TP53 *sequence variants identified

No	Exon	Nucleotide change	Amino acid change	Mutation classification	Previously reported?	No. of cases (*n *= 83)	Pathogenicity	Ethnicity
1	10	c. 1036G > T	p. E346X	Nonsense	Yes^1^	1	Deleterious	Malay

2	10	c. 1001_1006dup6	p. G334_R335dup	Duplication	Yes^2^	1	Likely deleterious	Malay

3	5	c. 413C > T	p. A138V	Missense	Yes^1^	1	Likely deleterious	Chinese

4	8	c. 853G > A	p. E285K	Missense	Yes^1^	2	Likely deleterious	Chinese

5	Intron 2	c. 74+14T > C	-	IVS	Novel	2	Likely benign	Chinese

6	Intron 3	c. 97-28T > A	-	IVS	Novel	1	Unknown	Chinese

7	Intron 6	c. 672+18G > C	-	IVS	Yes^1^	1	Likely benign	Malay

Mutation classification was made based on known databases or published studies.

^1^Reported in IARC *TP53 *database [18]. ^2^Ariffin *et al.*, 2008 [19]. IVS, intervening sequence

Notably of the five exonic *TP53 *mutation carriers, three had a family history of LFS-linked cancers, one had two cases of early-onset breast cancer in the family and one had no family history of cancer.

### Mutation c.1036 G > T (p. E346X)

**Figure 1:**
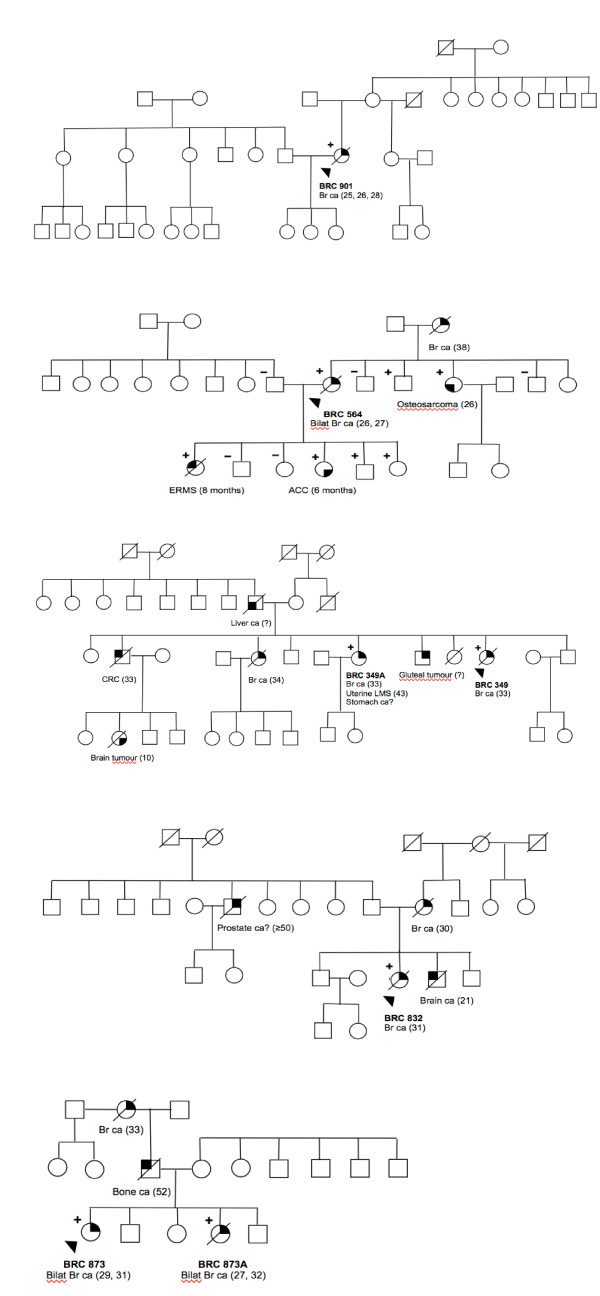
**Pedigrees of families with germline exonic mutations in *TP53***. Pedigrees of the following families: **(a) **Family BRC 901 with mutation *TP53 *p. E346X **(b) **Family BRC 564 with mutation *TP53 *p. G334_R335dup. **(c) **Family BRC 349 with mutation *TP53 *p. A138V **(d) **Family BRC 832 with mutation *TP53 *p. E285K **(e) **Family BRC 873 with mutation *TP53 *p. E285K. Index patients are indicated with an arrow. Individuals with cancer are indicated with filled symbol quadrants. Deceased individuals are indicated with a slash. Carriers with germline *TP53 *mutations are indicated with a "**+" **and non-carriers with a "**-"**. ACC, adrenocortical carcinoma; Br ca, breast cancer; Bilat Br ca, bilateral breast cancer, CRC, colorectal cancer; ERMS, embryonal rhabdomyosarcoma; Uterine LMS, uterine leiomyosarcoma.

This mutation was found in a Malay individual, BRC 901, with no reported family history of any cancer (Figure 1a). She was diagnosed with breast cancer at age 25, with recurrence in the same breast at age 26. At age 29, she developed Stage 4 contralateral breast cancer with metastasis to the bones, liver and lungs. This mutation, E346X, was found to be located within the tetramerization domain of *TP53 *and has previously been reported as a somatic mutation occurring in one tumor only (IARC *TP53 *database) [18].

### Mutation c.1001_1006dup6 (p. G334_R335dup)

This mutation was found in a Malay individual, BRC 564, whose family showed classic LFS features (Figure 1b). She presented with breast cancer at age 26 and contralateral breast cancer at age 27, and has one child diagnosed with embryonal rhabdomyosarcoma (ERMS) at eight months and another child with ACC at six months. The patient's mother was diagnosed with breast cancer at age 38 and her younger sister had osteosarcoma at age 26. Upon further analysis, it was found that this family was previously tested in the same hospital for *TP53 *mutations due to the presence of the childhood cancers. Further analyses have revealed two unaffected children and an unaffected brother as *TP53 *carriers, in addition to her two affected children and her younger sister with osteosarcoma [19].

This mutation, which is located within the *TP53 *tetramerization domain, involves a duplication of a 6 bp GGCGTG sequence, which results in an in-frame insertion of two amino acids (Arg and Gly), and is predicted to be likely deleterious.

### Mutation c.413 C > T (p. A138V)

This mutation was found in a Chinese individual, BRC 349, with LFL features (Figure 1c). The proband was diagnosed with breast cancer at age 32, with two sisters (BRC 349A and BRC 349B) affected by breast cancer at 33 and 34 years of age. Notably, one of her sisters, BRC 349A, was subsequently diagnosed with uterine leiomyosarcoma and stomach adenocarcinoma at 43 and 46 years of age, respectively, and was shown to have the same germline *TP53 *mutation. Her older brother was diagnosed with colorectal cancer at age 33 years, a younger brother was described by the family as having a tumor in the 'gluteal region', her father was affected with liver cancer at an unknown age and a niece was affected with a brain tumor at age 10.

**Figure 2:**
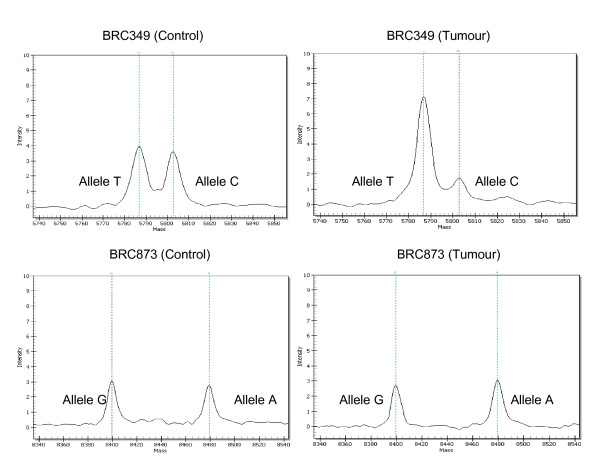
**Mass spectra of germline *TP53 *mutations p. A138V (c. 413C > T) and p. E285K (c. 853G > A) for LOH analyses**. **(a) **Loss of the wildtype C allele observed for sample extracted from FFPE breast cancer sample from BRC349 where 80% was tumor tissue **(b) **No LOH was observed for sample extracted from FFPE breast cancer sample from BRC873 where > 40% was tumor tissue. Left panel, control blood samples; Right panel, tumor samples; LOH, loss of heterozygosity.

The A138V mutation was found to be located within the DNA binding domain of *TP53*. This mutation has previously been reported as a somatic mutation in 51 tumors and, by *in silico *analysis with Align-GVGD, is predicted to be likely deleterious (IARC *TP53 *database) [18]. Notably, analysis of the DNA from FFPE breast cancer samples from BRC349 showed loss of the wild type *TP53 *allele (Figure 2a).

### Mutation TP53 c.853 G > A (p. E285K)

This mutation was found in two unrelated Chinese individuals (Figure 1d, e). The first patient, BRC832, was first diagnosed with breast cancer at 31 years, with a recurrence at 33 years. Her mother was diagnosed with breast cancer at age 30 and her brother with a brain tumor at age 20 years.

The second patient, BRC873, developed breast cancer at age 29 and contralateral breast cancer at age 31. The proband's younger sister, BRC 873A, developed bilateral breast cancer at the ages of 27 and 32, and was shown to have the same germline *TP53 *mutation. Other family history included her paternal grandmother being affected with breast cancer at 33 years of age and her father having bone cancer at 52 years.

The E285K mutation is located within the DNA binding domain of *TP53*. This mutation has previously been reported as a somatic mutation in 169 tumors and by *in silico *analysis with Align-GVGD, is predicted to likely be deleterious (IARC TP53 database) [18]. Haplotype analysis showed that these two families shared the same haplotype across the four microsatellite markers tested (data not shown). Analysis of the DNA from FFPE breast cancer samples from BRC873 showed that there was no loss of the wild type TP53 allele (Figure 2b)

### Intronic sequence variants

We have identified three intervening sequence mutations: c.74+14 T > C in two unrelated individuals, and c.97-28 T > A and c.672+18 G > C in one individual each. The mutation c. 672+18G > C was reported as a somatic mutation occurring in three tumors (IARC TP53 database) [18] whereas the remaining two mutations have not previously been reported.

### Case-control studies

None of the four exonic mutations identified in this study or the intervening sequence variant c.97-28 T > A, were found in any of the 880 unselected invasive breast cancer cases and 270 controls tested. Notably, c.74+14 T > C was found in 12/880 (1.4%) and 2/270 (0.74%) controls, and c.672+18 G > C was found in 14/880 (1.6%) breast cancer cases and 2/270 (0.74%) controls; suggesting that both variants are likely to be benign polymorphisms.

### Histopathological analysis

Breast cancers arising in germline *TP53 *mutation carriers were previously reported to be more likely to have amplification of HER2 as compared to non-*TP53 *carriers [20,21].

Of the seven breast cancer patients (five index and two family members) with germline exonic mutations in *TP53 *available for review, six have strong amplification of the HER-2 receptor (Table 3).

**Table 3:** Histopathological characteristics of breast cancers in individuals with *TP53 *germline mutations

Mutation	**Patient no**.	Age at diagnosis	Diagnosis	Histology	Grade	ER status	PR status	*HER-2 status*
p. E346X	BRC 901	25	Breast ca, L	IDC with DCIS	NA	-	+	+
		
		26	Breast ca, L	IDC	3	+	+	3+
		
		29	Breast ca, R	IDC	NA	NA	NA	NA

p. G334_R335dup	BRC 564	26	Breast ca, R	IDC	NA	NA	NA	NA
		
		27	Breast ca, L	IDC	3	-	+	3+

p. A138V	BRC 349	33	Breast ca, R	IDC	3	-	-	3+
	
	BRC 349A	33	Breast ca, L	IDC	2	-	-	-

p. E285K	BRC 832	31	Breast ca, R	IDC	2	-	-	3+
	
	BRC 873	29	Breast ca, R	IDC with DCIS	3	-	-	3+
		
		31	Breast ca, L	NA	NA	NA	NA	NA
	
	BRC 873A	27	Breast ca, L	NA	NA	NA	NA	NA
		
		31	Breast ca, R	Mucinous	2	+	+	3+

Breast ca, breast cancer; DCIS, ductal carcinoma *in situ*; ER, estrogen receptor; IDC, invasive ductal carcinoma; HER-2, human epidermal growth factor receptor 2; L, left; NA, not available; PR, progesterone receptor; R, right

## Discussion

Our study has shown that 5/83 (6%) of BRCA-negative Asian breast cancer patients diagnosed before age 35 years have germline exonic mutations in *TP53 *and that the average age of onset of breast cancer was 31 years. This is consistent with reports in other studies, where 0.8 to 5% frequency was reported among breast cancer patients diagnosed ≤ 40 years: in an clinic-based cohort of patients ≤ 40 years, 1/126 (0.8%) had a *TP53 *missense mutation [22]; in a Singaporean clinic-based cohort of breast cancer patients ≤ 35 years, 1/30 (3.3%) patients had a deleterious *TP53 *mutation [23]: in an Australian population-based cohort of two subgroups of early-onset breast cancer patients (≤ 30 years and 31 to 39 years), 5/94 (5.3%) had germline *TP53 *mutations [10].

Notably, in our study, three of the four families with LFS-linked cancers had *TP53 *germline mutations. Other genetics clinic-based testing showed that classical LFS criteria, LFL criteria or the Chompret criteria are useful in selecting families for *TP53 *testing. In a study of 180 Dutch families, *TP53 *mutations were found in 22/24 patients who fulfilled the Chrompret criteria and 18/24 patients who fulfilled either classic LFS or LFL criteria [9]. In a clinic-based study of a cohort of 525 individuals, 71/75 *TP53 *mutation carriers were shown to meet either classic LFS or Chompret criteria [8].

The majority of studies have been from clinical genetics units and recent breast cancer cohort studies suggest that not all germline *TP53 *mutation carriers meet either classic LFS or LFL criteria. Indeed, in our study, two out of five *TP53 *mutation carriers did not meet classic LFS or LFL criteria. Similarly, in an Australian study of 94 breast cancer patients, only two out of five carriers met either classic LFS or LFL criteria [10] and in a UK-based study of 100 breast cancer patients, two out of four carriers met these criteria [11].

Our study has identified four exonic mutations (A138V, E285K, E346X and p. G334_R335dup), three of which have not previously been identified as germline mutations. Through co-segregation and LOH studies, we show that A138V and E285K are likely to be pathogenic. In addition, we show that E285K is recurrent in two unrelated Chinese families. Other recurrent mutations have previously been reported, including the *TP53 *R337H mutation in southern Brazil [16] and the M133T mutation found in two unrelated African-American families [24], but to the best of our knowledge, this is the first report of a recurrent *TP53 *mutation in an Asian population.

In addition, our study has also identified three intronic variants (c. 74+14 T > C, c. 97-28 T > A and c. 672+18 G > C), of which c. 74+14 T > C and c. 672+18 G > C are benign as we show that they are also found in non-cancer control individuals.

Notably, in our cohort of early-onset breast cancer patients, 11%, 6% and 5% had germline mutations in *BRCA1, BRCA2 *and *TP53 *respectively. Given the comparable frequency of *BRCA1, BRCA2 *and *TP53 *mutations among patients diagnosed at ≤ 35 years and the high penetrance of *TP53 *mutations, we suggest that all patients who develop breast cancer at ≤ 35 years of age should be offered genetic counseling and a family history of LFS-linked cancers should be carefully obtained. Our findings are consistent with the recommendation by the National Comprehensive Cancer Network (NCNN) that all breast cancer patients who are diagnosed before the age of 30 and are BRCA-negative should be offered genetic counseling and testing for *TP53 *[25].

In addition, our data show that HER2 amplification may be a useful marker in identifying *TP53 *germline carriers, as six of the seven breast tumors (86%) in *TP53 *germline mutation carriers had amplification of *ERBB2 *(HER2). Previous reports found that 20/29 (69%) and 10/12 (83%) of breast tumors from germline *TP53 *carriers had HER2 amplification or over-expression [20,21] and having a HER-amplified tumor increased the odds of having *TP53 *germline mutation by nearly seven-fold [21]. These data are consistent with the observation that somatic mutations in *TP53 *are found in the majority of HER2-amplified tumors, but the mechanism of interdependence between *TP53 *and HER2 require further work [20,21].

## Conclusions

In conclusion, we show that *TP53 *germline mutations occur at a comparable frequency with *BRCA1 *and *BRCA2 *germline mutations among Asian early-onset breast cancer patients, and occur predominantly, but not exclusively, among families which have a family history of LFS-linked cancers.

## Abbreviations

ACC: adrenocortical cancer; BRCA1: breast cancer 1 gene; BRCA2: breast cancer 2 gene; ERMS: embryonal rhabdomyosarcoma; FFPE: formalin fixed paraffin-embedded; HER2: human epidermal growth factor receptor 2; LFL: li-fraumeni-like; LFS: li-fraumeni syndrome; LOH: loss of heterozygosity; MyBrCa: Malaysian Breast Cancer Study; NCNN: National Comprehensive Cancer Network; TP53: tumor protein 53.

## Competing interests

The authors declare that they have no competing interests.

## Authors' contributions

YSY, HA, TMK, CKF, NAMT and YCH recruited patients, collected clinical data and YSY, HA, TMK, NAMT and YCH were responsible for genetic counseling of patients. DSCL and LML conducted pathological analyses, including LOH studies. DSCL, PK, KIN and KS were responsible for laboratory analyses, including sequencing, genotyping and haplotype analyses. DSCL and TSH conceived the study and wrote the manuscript. All authors read and approved the final manuscript.
